# The Iterative Mindset Method: a neuroscientific theoretical approach for sustainable behavior change and weight-loss in digital medicine

**DOI:** 10.1038/s41746-023-00910-y

**Published:** 2023-09-26

**Authors:** Kyra Bobinet, Stephanie M. Greer

**Affiliations:** 1 Fresh Tri, Inc., 13001 East Zayante Road, Felton, CA 95018 USA; 2 SMG Labs, LLC, 2621 Judah Street, San Francisco, CA 94122 USA

**Keywords:** Technology, Weight management, Social neuroscience

## Abstract

With the growing prevalence of chronic conditions driving 85% of all healthcare costs, digital health offers a promising opportunity to reverse disease and improve health at-scale. The healthcare industry’s predominant approach to behavior change is performance-based with a focus on goals and tracking. This has not reversed the epidemic of chronic diseases and also can harm chronically ill and vulnerable patients via perceived failure-induced loss of motivation. Still nascent, the digital health industry is uniquely positioned to adopt and scale new and better behavior change approaches. In this paper, we present the theoretical foundation and initial findings of a neuroscience-based behavior change approach—what we call the Iterative Mindset Method^TM^. We discuss its promise, as a potentially more effective, neuroscience-based approach to changing health behaviors long-term, particularly in vulnerable populations. We conclude with avenues for future research.

## Background

More than 85% of all healthcare costs, including 99% of Medicare and 83% of Medicaid dollars spent, are driven by chronic conditions^1^. In addition, all chronic conditions aside from hyperlipidemia and COPD have increased in prevalence over the past 25 years^2^. As chronic conditions are now the leading cause of death, disability^3^, and loss of productivity^4^, reducing their incidence could generate significant gains in quality of life, economic growth, and societal value. Indeed, the booming digital health industry predominantly targets chronic conditions. With a predicted Compound Annual Growth Rate of 14.5% for 2019–2025^5^ and a record-setting $29.1B invested in 2021^6^, digital health offers unprecedented potential to impact chronic disease.

However, digital health must produce sustainable outcomes to become a valuable mainstay of healthcare. The adoption of long term healthy lifestyles, comprised of good habits in nutrition, exercise, sleep, mental well-being, and social support, is the most significant opportunity to lower the prevalence of chronic conditions and improve health at scale^7^. To reverse such trends of chronic conditions, a methodology capable of lasting behavior change must be characterized and adopted writ large.

Yet, to date, lowering the incidence of chronic conditions via ingraining healthy behaviors has eluded the healthcare industry. This may lie in the predominant methods used to facilitate behavior change, which promote a performance mindset^8^. This type of mindset hails from the *performance goal orientation*, which is defined as “…seek[ing] to gain favorable judgments… or avoid negative judgments of [one’s] competence” or ability^9^. In contrast, a *learning goal orientation* is defined as “[the] desire to develop the self by acquiring new skills, mastering new situations, and improving one’s competence.”^10^ In most current digital health applications, a plethora of performative goal-setting and tracking features (e.g., SMART goals, calorie-, carb-, or steps-counting) as well as competitions and challenges (e.g., a 21-day challenge, leaderboards, or team-based competitions) are used to motivate users to change their behavior and successfully achieve their goals. Even the National Diabetes Prevention Program Lifestyle Change Program (DPP), which aims for long-term healthy behavior change, uses performative tools such as weekly weight and activity tracking as well as food logging, historically.

While sometimes initially effective, a performance mindset becomes harmful in several important ways. Performance mindsets reduce the value placed on effort, practice, and hard work^11^ and can increase trait (or stable) anxiety, fear of failure, and cheating to avoid failure^11^. Also, performance mindsets succeed only for those with already high self-efficacy, and only when the rules of the task are consistent^11^. Finally, and most importantly, performance mindsets cause individuals to define their success using measurable and highly specific outcomes (Table 1).

**Table 1:** Sample statements representing Iterative Mindset language in sentiment analysis.

Sample statements	“I didn’t put on the weight overnight, I’m not going to lose the weight overnight.”
	“Victim mentality says that there’s nothing you can do, but there is! You just have to try more than the day before.”
	“You can always find an alternative, you can always do something.”
	“Experiment! If you don’t like what you are eating, you are not going to be very successful.”
	“I’ve overcome it once, I know how to get through it.”

However, many health goals by their nature are reversible (e.g., losing, but then regaining, 20 lbs), compared to other goals which are irreversible (e.g., graduating with a diploma)—and this potentiates failure in the long-term even after initial success. By evidence of the diet industry’s 80% weight recurrence rate^12^, a vast majority of people are “failing” from using this type of mindset—either by never reaching their goal at all or achieving it temporarily and then relapsing.

For the brain, these repeated failures are not a benign event. One brain area, the habenula, activates when one thinks they failed (even subconsciously) and downregulates motivation to try again^13^. Thus, the person quits trying to change, ruminates in self-blame, and becomes frozen in a state of what is termed in the literature as ‘learned helplessness’^14^. Additionally, recent research on the habenula is producing mounting evidence that it serves as a key locus for depression, anxiety and even addiction in the brain^15–17^—all comorbidities that make behavior change even more difficult. Although performance mindsets may be effective for those who are already expert at being healthy, for 60% of Americans who have chronic conditions, including the 20% of high-risk patients who consume 80% of all healthcare costs^18^, performance mindsets are unlikely to lead to lasting change and may even be harmful.

**Fig. 1:**
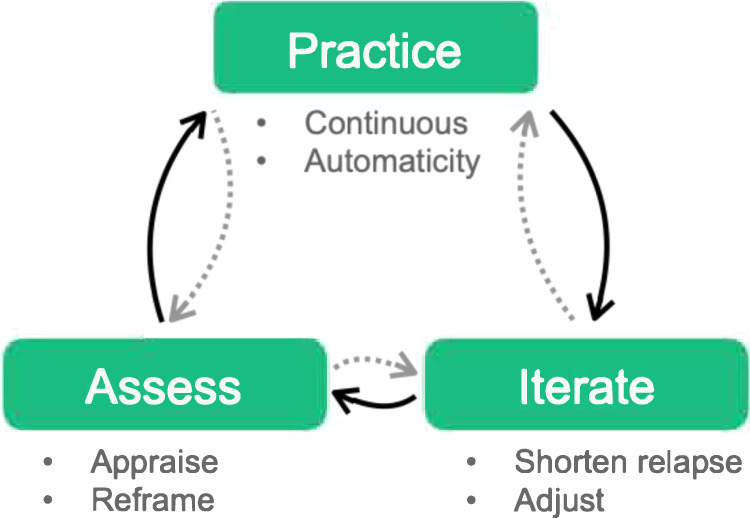
Iterative Mindset Method. The Iterative Mindset Method contains three variables of Assess, Practice, and Iterate, each of which having two defining components. Practice includes both attaining continuous practice and achieving automaticity of a habit as a result of continuous practice. Iterate includes an emphasis on shortening relapses through making adjustments and tweaks in order to prevent or recover from disruption in one’s practice. Assess entails an appraisal of what one has learned as well as determining what is next (to try, to practice, etc.).

Thus, as an alternative to a performance-mindset approach, in the current work, we developed a neuroscience-based theoretical approach for sustainable behavior change in digital medicine—The Iterative Mindset Method™ (IMM). The IMM involves a dynamic cycle between three variables: practice, assessment, and iteration (Fig. 1). The potential for lasting (rather than temporary) change using the IMM comes from the IMM’s emphasis on supporting the individual in continuous practice—with automaticity of habits as the explicit purpose of effort. This emphasis means that new behaviors are meant to become less effortful and more automatic and intrinsically motivating over time without the need for continued rewards or explicit memory systems.

Starting with *practice*, for a particular individual or timepoint, the IMM cycle can move in either direction among the three variables as well as cycle between two of the variables before continuing to the third. As an individual continuously practices, they may also move into *iteration*, which is a process of adjusting one’s practice to make it better, easier and/or more fitting. Such experimentation and adaptation potentiate a practice to become an automatic, lasting habit through shortened relapses and persistent repetition. The third component, *assess*, includes (1) an implicit appraisal of how well a practice fits into an individual’s lifestyle/routines/personality as well as (2) any reframing needed to judge all experience as progress and not failure.

Altogether, this dynamic, non-linear cycle offers a method for lasting behavior change that is adaptive, self-renewing, and perpetual. The IMM enables automaticity of habits through repeated continuous practice. It protects from failure, and conceivably habenula activation, through iteration on one’s effort and shortening relapse periods. This is supported by data from the National Weight Control Registry showing that those who have the shortest relapse periods are the ones who achieve lasting weight loss^19^. Finally, the IMM uses assessment to both appraise the efficacy of one’s practice or iteration, as well as reframe any perceived failure into something beneficial.

The IMM improves on other theories, including related mindset and learning-orientation research, in three distinct ways: (1) IMM is both belief and action-oriented rather than belief alone, (2) IMM explicitly focuses on combining experimentation and adaptation with circumvention and reframing of failure to maintain motivation and perpetual effort, and (3) IMM focuses on the process, rather than the product, of behavior change.

The following sections highlight some intriguing preliminary data, from three independent samples, indicating the potential of the IMM to impact behavior change and long-term weight loss. We outline remaining empirical questions and conclude with a discussion of applications for digital health.

### Evidence

First, we conducted 2 years of nationwide research on low wage earners for a jumbo U.S. retailer. Namely, we collected more than 200, 1-h qualitative employee interviews (geographically distributed and demographically representative of the U.S. population) using a standardized interview instrument. We focused our analyses on a small subset (*n* = 51) who reported losing a significant % of their body mass and maintained it for two or more years.

We conducted a qualitative analysis to see if these 51 participants reported using an iterative approach. Specifically, we engaged participants in semi-structured interviews starting with a core question and then incorporated related follow-up questions. We also prompted participants with, “then what” to obtain additional information. Two people conducted all conversations online via audio interviews. We then listened to the responses to investigate the presence of certain words, themes, and concepts. We followed inductive approaches for data analysis developed to help link theoretical ideas^20,21^. Following procedures of grounded theory, we conceptualized latent patterns^22,23^. In the first step of the coding, we noted broader statements related to key components of an iterative approach for lasting behavior change. These included (1) approaching behavior change as a practice, “trying”, or an experiment, (2) reframing failures, and (3) adjusting when reaching an impasse (e.g., a failed launch, boredom, relapse, or need to ‘level-up’). In the second step, axial coding, we identified connections and assigned labels to first-order concepts and then situated these into clearly delineated themes. Then, going back and forth between primary data and emergent ideas, and drawing on existing theory, we refined the overarching categories. The first author identified an organizing framework with three clear aggregate dimensions, which are labeled practice, assessment, and iteration (see Fig. 1). Taken together, this iterative approach was the only common pattern across the 51 participants who maintained weight loss long-term. Indeed, and in line with past research^24^, mere demographics (e.g., sex, race) and programmatic similarities (e.g., joining a weight loss group) failed to reveal clear patterns. The main commonality among those who lost a meaningful amount of weight and kept it off was their iterative method.

**Fig. 2:**
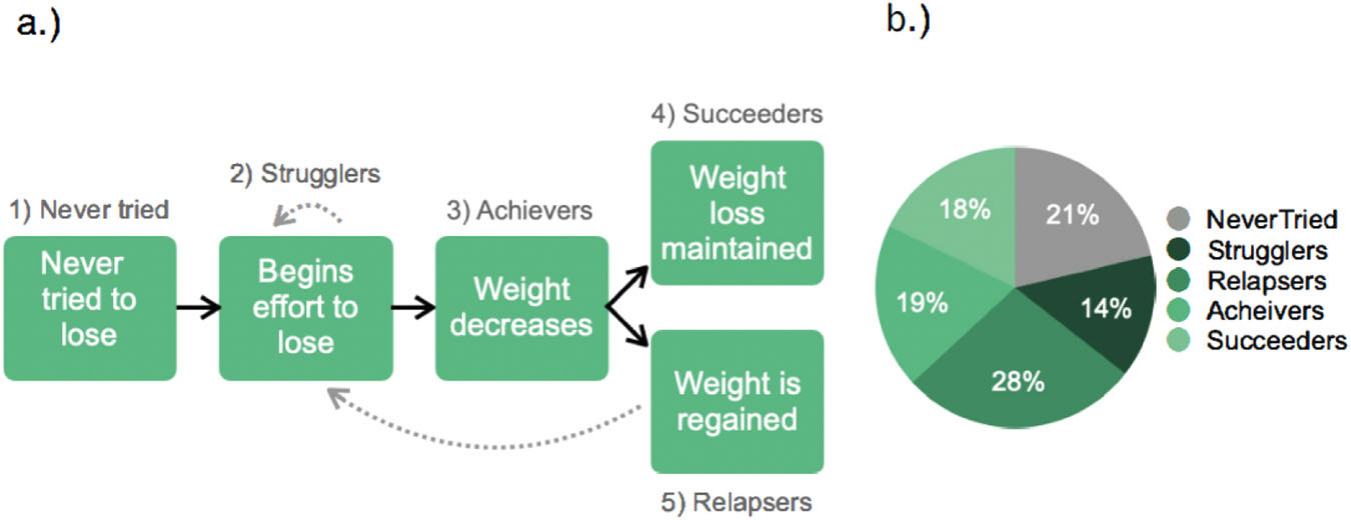
Weight loss journey segmentation. **a** Weight loss journey segmentation diagram and **b** prevalence of weight loss journey segmentations in a general population sample.

**Fig. 3:**
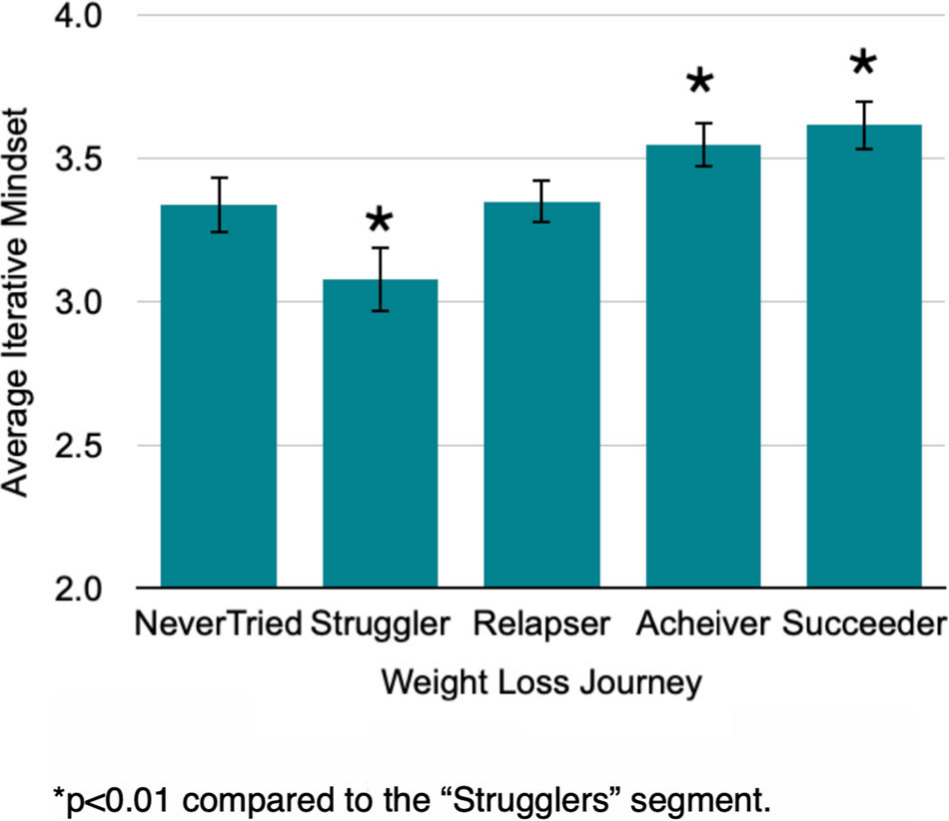
Iterative Mindset by segment. Data from 821 survey respondents from the general population. Two-tailed *t*-test with samples of unequal variance used to generate *p*-values. Error bars represent 95% confidence intervals.

Second, building on these qualitative findings, we developed a set of questions to assess if participants used an iterative approach and if this correlated with weight loss. In a sample of 2600 participants, we first identified four mutually exclusive weight loss segments, or journeys, that we named Strugglers, Relapsers, Achievers, and Succeeders (Table 2 and Fig. 2). We used this pilot work to inform our correlation study. Namely, we surveyed 821 individuals and asked them to complete the IMM and weight loss profile assessment. Specifically, for the IMM measures, we asked participants to report if they engaged in practice (e.g., one less soda daily), reframing (e.g., learning what doesn’t work) and/or adjusting and adapting their practice (e.g., switching up to diet soda when ‘one less soda daily’ doesn’t work). For weight loss profiles, we asked one question related to each profile (e.g., I have lost weight in the past but regained it all—Relapser). As shown in Fig. 3, those who struggled to lose weight without real success (Strugglers) were the least likely to use an iterative approach, relative to any other group (*p* > 0.001). Those in the Succeeder group, maintaining weight loss over 2 years, were the most likely to report using an iterative method, with statistically significantly higher levels as compared to all other groups, other than the Achievers (*p* < 0.01).

**Table 2:** Weight loss journey behavioral segmentation question and responses.

Q: Which of these most closely describes your experience with losing weight?	Segment
I have lost weight and kept it off for more than 2 years.	Succeeder
I am currently losing weight but have kept it off less than 1 year.	Achiever
I have lost weight in the past but regained it all.	Relapser
I have tried to lose weight with no success.	Struggler
I have never tried to lose weight.	Never Tried

Third, in another unique sample (*n* = 43), we investigated if it might be possible to alter or train an iterative approach through a digital program. In this longitudinal pilot study, all participants were low wage earners and thus at-risk for chronic conditions^25^. We first administered pre-test assessments, with a focus on the iterative method. Immediately following pre-tests, we delivered the iterative intervention. Participants had access for 60 days to a habit formation app designed to support an iterative approach. For example, the app guided the user to outline a habit to practice daily and there were also weekly online education sessions about iteration conducted by a registered dietician/health coach. At follow-ups, in addition to assessing the iterative approach, we also administered the Self-Report Habit Index Scale (SRHI), including an Automaticity subscale^26^. We also assessed weight at pre-test and 60-day follow-up to investigate weight loss.

**Fig. 4:**
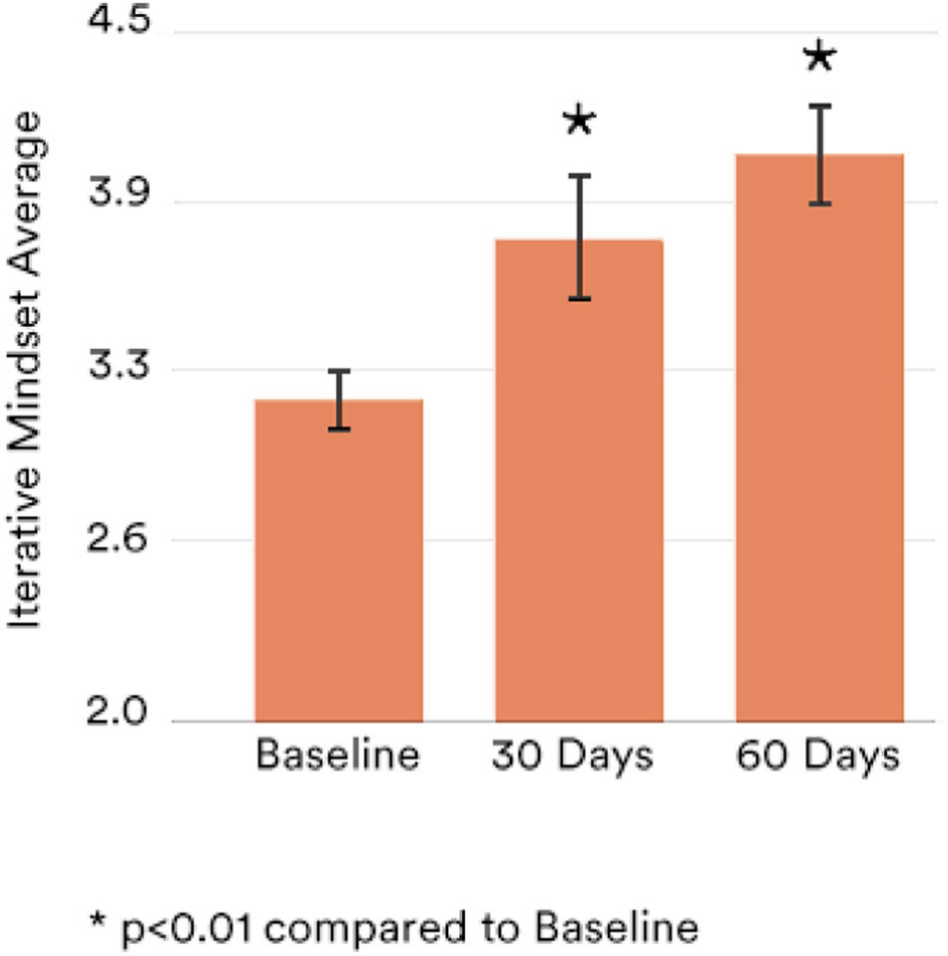
Study 3 longitudinal iterative intervention data: iterative mindsets. Iterative Mindset proves trainable as part of a 60-day weight loss intervention in a vulnerable population that resulted in weight loss of ~1 lb/week, *p* < 0.01. Data was collected from a prospective 60-day digital weight loss pilot study of an Iterative Mindset Method intervention for 97 LWE participants, with 43 completers. Two-tailed *t*-test with samples of unequal variance used to generate *p*-values. Error bars represent 95% confidence intervals.

**Fig. 5:**
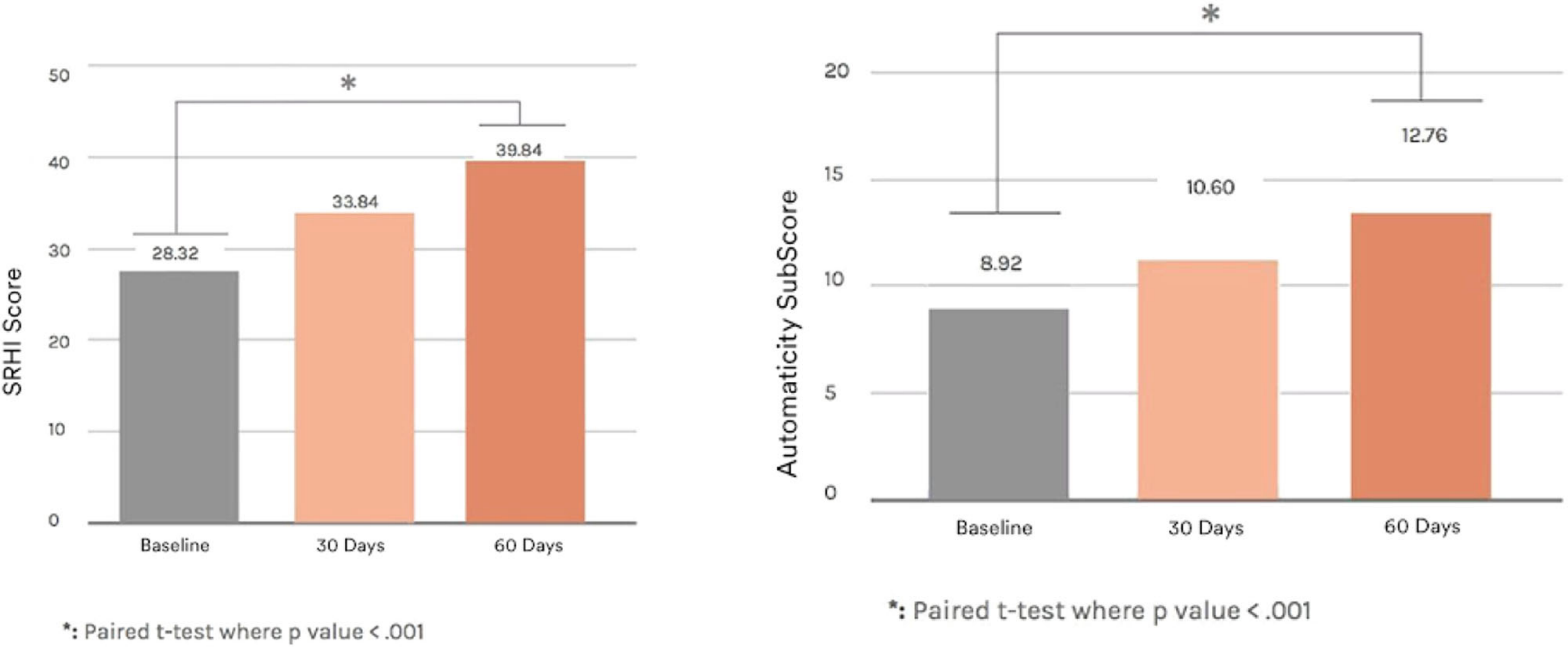
Study 3 longitudinal iterative intervention data. SRHI Self-Report Habit Index.

As shown in Fig. 4, over the course of the intervention, the average IMM scores increased above baseline levels by 1.16 standard deviations. Participants also reported statistically significant increases in both habit formation and automaticity scores, indicating that IMM may help with habit formation (see Fig. 5)^27^. Finally, participants also lost 2.76% body weight on average, a statistically significant decrease (*p* < 0.01). These data show initial evidence that the IMM is trainable and may be a possible leverage point for behavior change and weight loss^28^.

## Discussion and future directions

Overall, the goals of the current work were twofold. First, we provided a strong theoretical foundation, including the neuroscience reasoning for the IMM. Second, we provided initial evidence from three different studies and samples using a multi-method approach, including qualitative, correlational, and longitudinal designs. The findings converge on a common theme: iteration is a key component of sustained weight loss.

The theoretical foundation in conjunction with our initial pilot data provides a springboard for future inquiry. Namely, we encourage a systematic line of work that first validates an assessment of Iterative Mindsets, establishing both validity and reliability. Then, future research can use this assessment to examine behavior change over time and to start to answer questions related to the origin and development of an Iterative Mindset. This psychometric foundation paves the way for randomized control studies that articulate processes of change and boundary conditions. We highly recommend that this work takes a heterogeneity attuned approach—one that outlines the contextual factors that must be in place for interventions to work reliably^29^.

## Conclusion

Digital health shows great promise but risks repeating the incumbent diet industry’s trend of creating short-term results, high relapse rates, and harm to more vulnerable populations—if it continues to rely upon performative methods. While performance mindsets and tools may drive results for high-achieving, high self-efficacy individuals (also statistically likely to be at the top of health, education, and income segments), it can be dangerous for those who do not possess these advantages^30^. Nascent, emerging neuroscientific evidence points to the habenula as a potential locus for motivation loss and depression, and therefore subsequent relapse and reversal of initial performance-based results. Further studies are needed to elucidate the relationship(s) between habenular action and long-term health behavior change.

In the current work, we suggested replacing performative tools in digital health with a more iterative and process-focused approach. This iterative method provides a critical innovative way to improve chronic conditions through designing more effective and inclusive digital health solutions. It can also foster perpetual healthy effort, potentiate habit formation, protect from perceived failure, improve resilience and ultimately lead to lasting weight loss. Overall, we hope our initial theorizing and evidence sparks interest in solidifying the transformative potential of the IMM in digital health.

### Reporting summary

Further information on research design is available in the Nature Research Reporting Summary linked to this article.

### Supplementary information

Reporting Summary

## Data Availability

The data that support the findings of this study are not publicly available due to privacy, commercialization, and/or ethical restrictions. However, data can be made available upon request from the corresponding author.
